# Rapid induction of clinical remission in SAPHO syndrome using high-dose Tripterygium glycosides: A case report

**DOI:** 10.1097/MD.0000000000021102

**Published:** 2020-07-02

**Authors:** Liang Gong, Lun Wang, Yihan Cao, Chen Li

**Affiliations:** a Institute of Clinical Medicine; b Department of Radiology; c Department of Traditional Chinese Medicine, Peking Union Medical College Hospital, Chinese Academy of Medical Sciences and Peking Union Medical College, No. 1 Shuaifuyuan, Beijing, China.

**Keywords:** remission induction, SAPHO syndrome, Tripterygium wilfordii hook f

## Abstract

**Rationale:**

Synovitis, acne, pustulosis, hyperostosis, and osteitis (SAPHO) syndrome is a rare disease without standard treatments. Tripterygium wilfordii hook f (TwHF) is a traditional Chinese herb with anti-inflammatory effect, and 1.0 mg/(kg·d) dose of Tripterygium glycosides has been reported to significantly improve the disease activity of a SAPHO patient in a case report. However, the optimal dose of TwHF is still unclear. Here, we report the first case of SAPHO patient who achieved rapid remission in clinical symptoms after receiving 1.5 mg/(kg·d) dose of Tripterygium glycosides treatment.

**Patient concerns:**

A 67-year-old woman noted palmoplantar pustulosis and pain in the anterior chest wall and waist. Bone scintigraphy demonstrated the typical tracer accumulation feature and magnetic resonance images showed bone marrow edema in lumbosacral vertebra.

**Diagnoses:**

The diagnosis was made by dermatological and osteoarticular manifestations and classical signs in bone scintigraphy in accordance with the diagnostic criteria proposed in 2012.

**Interventions:**

Tripterygium glycosides was given with a primary dose of 1.5 mg/(kg·d) for 1 month and then reduced at a rate of 10 mg every 2 weeks until 1.0 mg/(kg·d) for a long-term maintenance.

**Outcomes:**

Fast-induced remission on clinical manifestations was achieved and magnetic resonance imaging abnormality was improved significantly. Additionally, no apparent side effects were observed.

**Lessons:**

1.5 mg/(kg·d) dose of Tripterygium glycosides seems to have fast-induced remission than 1.0 mg/(kg·d) with reliable safety. Besides, Tripterygium glycosides may also have a pharmacological effect of inhibiting osteolysis and enhancing bone strength.

## 1 Introduction

Synovitis, acne, pustulosis, hyperostosis, and osteitis (SAPHO) syndrome is a rare disease, characterized by osteoarticular manifestations and skin lesions.^[1]^ It is generally believed that the pathogenesis of SAPHO syndrome is related to genetic, infectious, and immunologic factors.^[2]^ The increase of proinflammatory cytokines such as IL-6 and IL-8, the imbalance of the ratio between Th17 and regulatory T cells, and the decrease of TGF-β1 contribute to the pathogenesis of SAPHO syndrome.^[3–5]^ Treatments for SAPHO syndrome include nonsteroidal anti-inflammatory drugs (NSAIDs), antibiotics, corticosteroids, bisphosphonates, immunosuppressants, conventional synthetic disease-modifying antirheumatic drugs (csDMARDs), biological agents like TNF-α inhibitors.^[5]^ However, there is no standard treatment for the disease.

Tripterygium wilfordii hook f (TwHF) is a traditional Chinese herb with anti-inflammatory effect, which has been used in the treatment of rheumatoid arthritis, ankylosing spondylitis, and other immunologic diseases.^[6,7]^ Tripterygium glycosides tablet is the most commonly used TwHF drug in the clinic. The regular therapeutic dose of Tripterygium glycosides is 20 mg 3 times per day, which was selected based on the dose of 1.0 mg/(kg·d). Our team has reported a case of remarkable remission of SAPHO syndrome in response to 1.0 mg/(kg·d) dose of TwHF.^[8]^ However, the recommended dose of Tripterygium glycosides is 1.0 to 1.5 mg/(kg·d); therefore, whether a higher therapeutic dose is still safe and can lead to more significant and comprehensive improvement of SAPHO is currently unclear. Here, we report the first case of SAPHO patients who achieved significant remission without apparent side effects in a short period of time after receiving 1.5 mg/(kg·d) dose of Tripterygium glycosides treatment.

## 2 Case report

Written informed consent was obtained from the patient for publication of this case report, and the study was approved by the Ethics Committee of Peking Union Medical College Hospital and the Chinese Academy of Medical Sciences.

A 67-year-old woman noted palmoplantar pustulosis (PPP) in November 2017. Thereafter, the skin lesions suffered repeated remission and occurrence, but aggravated gradually. Four months later, the patient presented with pain in the anterior chest wall and waist. Although the bone pain could be relieved after receiving NSAIDs, the symptoms relapsed with pain and movement restriction in the lumbar region. The patient was admitted to our hospital in November 2018, and there was no family history of similar symptoms reported.

**Figure 1:**
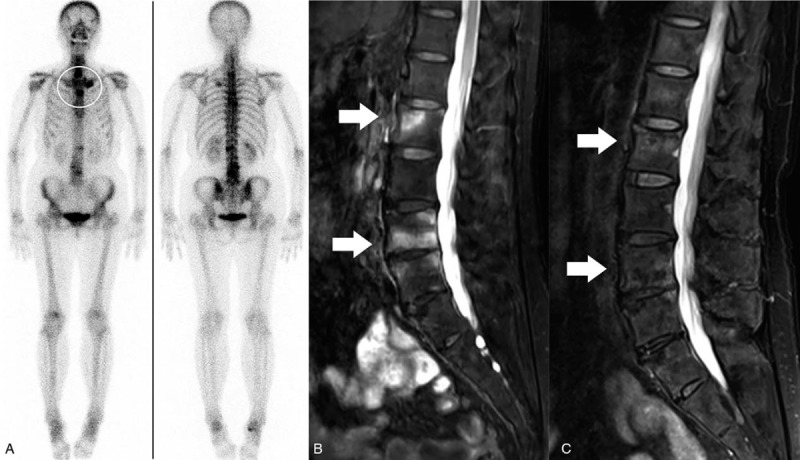
A, ^99m^Tc-MDP whole body bone scintigraphy showed the characteristic “bull's head” sign in the anterior chest wall (white circle). B, MRI (T2 fat suppression sequence) revealed the high signal intensity (white arrow), demonstrating bone marrow edema. C, Bone marrow edema disappeared (white arrow) after the treatment of Tripterygium glycosides. MRI = magnetic resonance imaging.

On admission, laboratory examination showed an elevation of hypersensitivity C-reactive protein (hsCRP) 6.87 mg/L and erythrocyte sedimentation rate (ESR) 36 mm/h. Blood test, liver, and kidney functions were within the normal range. Her rheumatoid factor, antinuclear antibody, and human leukocyte antigen-B27 were all negative. Whole body bone scintigraphy via ^99m^Tc-MDP showed increased radionuclide uptake in the sternum, left first anterior rib, and lumbar vertebrae, demonstrating a characteristic “bull's head” sign of SAPHO syndrome (Fig. 1A). Magnetic resonance images (MRI) showed multiple patchy slightly long T1 and long T2 signals in lumbosacral vertebrae, and the T2 fat suppression sequence showed high signal intensity, indicating bone marrow edema (Fig. 1B). Based on her clinical manifestations, imaging results, and bone scintigraphy, the patient was diagnosed with SAPHO syndrome according to the diagnostic criteria proposed by Nguyen et al in 2012.^[9,10]^

The patient weighed nearly 60 kg and was treated with oral Tripterygium glycoside tablets 30 mg 3 times a day based on a dose of 1.5 mg/(kg·d). After 1 month, the dose was reduced by 10 mg every 2 weeks and finally reduced to 1.0 mg/(kg·d) for long-term maintenance.

**Figure 2:**
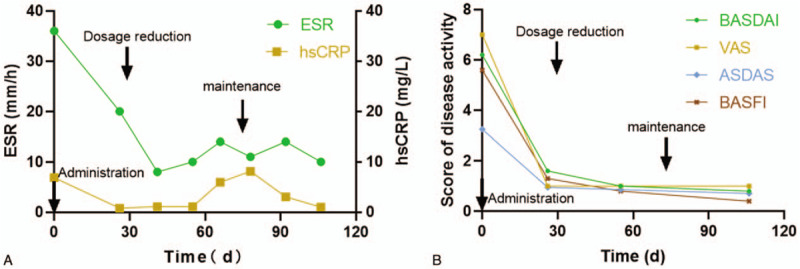
A, B, Improvement of inflammatory indicators, decrease of disease activity scores after the treatment of Tripterygium glycosides. The black arrows indicate the administration, dosage reduction, and maintenance treatment respectively.

**Figure 3:**
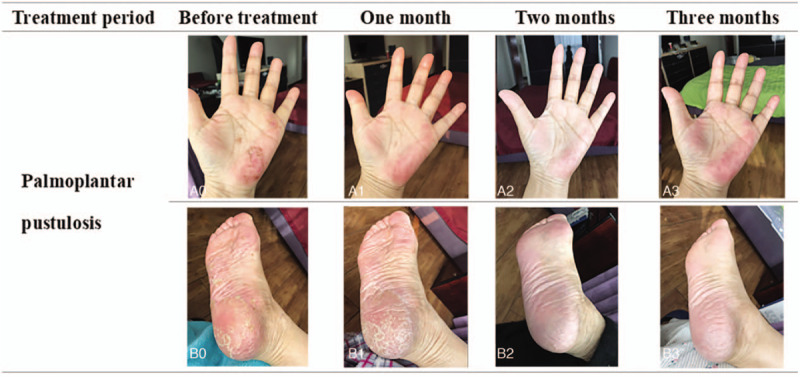
The dynamic change of palmoplantar pustulosis after administration of Tripterygium glycosides. A, B, Palm and plantar manifestations (0) before treatment, (1) one month later, (2) two months later, (3) three months later. The original pustules in the palms and soles of the feet disappeared gradually.

After the treatment, the bone pain and disease activity of the patient were significantly relieved, showed by the decrease of Visual Analog Scale, Bath Ankylosing Spondylitis Activity Index, Bath Ankylosing Spondylitis Functional Index, and Ankylosing Spondylitis Disease Activity Score (Fig. 2B). Her PPP improved significantly that the original lesions disappeared and no new pustules were found (Fig. 3). The inflammatory indicators like hsCRP and ESR decreased significantly after 1 month of treatment, although there were some fluctuations with the decrease of drug dosage, they remained within the normal range during the period of maintenance treatment (Fig. 2A). In terms of bone metabolism, serum C-terminal telopeptide of β-I collagen (β-CTX) decreased from 0.699 to 0.39 ng/L, and serum osteocalcin increased from 1.12 to 1.82 ng/mL. After 3 months of treatment, the intensity of bone marrow edema in the lumbosacral vertebrae on MRI was reduced (Fig. 1C).

During the whole follow-up, the patient strictly complied with the drug treatment regimen. There were no abnormalities in routine blood tests and laboratory examinations of hepatic and renal function, and no significant adverse events such as gastrointestinal disorders, fatigue, headache, or dizziness were observed. As the patient is a menopausal woman, reproductive toxicity was not concerned.

## 3 Discussion

This is the first case report on 1.5 mg/(kg·d) dose of Tripterygium glycosides in the treatment of SAPHO syndrome. The changes in clinical symptoms, disease activity score, and serological inflammatory factors showed that the disease activity significantly decreased in a short period of time after treatment and could be maintained at a low level during the follow-up. Additionally, no apparent side effects such as gastrointestinal disorders, fatigue, headache, or dizziness were observed.

Compared to the previously reported case of 1.0 mg/(kg·d) TwHF treatment, the present case could add 3 new findings. First, PPP as a major skin manifestation of SAPHO patients could also be significantly improved by Tripterygium glycosides, which is meaningful for the life quality and overall prognosis of SAPHO patients. Second, 1.5 mg/(kg·d) dose of Tripterygium glycosides seems to have faster-induced remission than 1.0 mg/(kg·d). Third, in addition to the mechanism of anti-inflammation, Tripterygium glycosides may also exert beneficial effects through the regulation of bone metabolism.

94.6% of SAPHO patients suffer from cutaneous lesions, mainly manifested as PPP, followed by severe acne.^[11]^ PPP is a chronic recurrent skin disease with eruption of sterile pustules on palms and soles. Patients with PPP usually present with pain or itching, and there is a significant correlation between disease severity and quality of life.^[12]^ The improvement of skin lesions is meaningful for SAPHO patients to get a better quality of life and overall prognosis.

Previous studies have found that the anti-inflammatory efficacy of TwHF is dose-dependent,^[13,14]^ and according to our own clinical experience and observation, 1.5 mg/(kg·d) dose of Tripterygium glycosides might have faster-induced remission than 1.0 mg/(kg·d). The previously reported case receiving 1.0 mg/(kg·d) treatment achieved maximum remission after 3 months, and maintained at a low-level inflammatory state with the concomitant use of MTX. In the present case, the patient achieved comparative maximum remission only after 1 month, and such improvement could be maintained during the follow-up without apparent adverse effects. However, given the heterogeneity of SAPHO patients, the difference in efficacy and toxicity between these 2 doses of Tripterygium glycosides needs to be confirmed by further prospective research. Considering that high-dose exposure may also lead to greater hepatotoxicity,^[15]^ nephrotoxicity,^[16]^ and reproductive toxicity,^[17]^ 1.5 mg/(kg·d) dose of Tripterygium glycosides should be prescribed to patients with normal hepatic and renal function and no desire to have children. To avoid irreversible damages, the hepatic and renal function should be tested regularly during the treatment period, and for premenopausal female patients, their menstrual condition and, if necessary, hormone levels should also be monitored.

The inflammatory response induced by infection or others is closely related to bone metabolism. The inflammation characterized by the T cells activation and cytokines release can lead to the imbalance of ratio between Receptor activator nuclear factor κ B ligand and osteoprotegerin, and such abnormal bone metabolism will eventually result in bone destruction.^[18]^ In this case, 1.5 mg/(kg·d) dose of Tripterygium glycosides not only play an anti-inflammatory role in improving osteoarticular pain and skin lesions, but also showed benefits in bone metabolism. Osteocalcin and β-CTX are common biochemical markers for bone metabolism. Osteocalcin is a vitamin K-dependent calcium-binding protein, which is mainly synthesized by osteoblasts and reflects the activity of osteoblasts and bone turnover. β-CTX is a decomposition product of type I collagen, which is used to measure the activity of osteoclasts and bone resorption. A retrospective analysis showed that the main mechanism of bone destruction in SAPHO syndrome was the enhancement of bone resorption, and the level of β-CTX could reflect the disease activity of SAPHO syndrome.^[19]^ After the 1.5 mg/(kg·d) Tripterygium glycosides treatment, the increase of serum osteocalcin accompanied by the decrease of serum β-CTX, suggested that Tripterygium glycosides may also have a pharmacological effect of inhibiting osteolysis and enhancing bone strength, which is consistent with the study of Huang et al.^[20]^

Compared with other treatments, Tripterygium glycosides has the advantage of lower cost and more comprehensive efficacy; therefore, it should be widely recommended for the treatment of SAPHO syndrome. In addition, the combination of Tripterygium glycosides with other csDMARDs or biological agents might achieve a better therapeutic effect.

## 4 Conclusion

This case suggests that through the mechanism of anti-inflammation and bone metabolism regulation, 1.5 mg/(kg·d) dose of Tripterygium glycosides had reliable safety and could lead to significant and rapid remission on both osteoarticular and cutaneous lesions. Therefore, it is necessary to design further study to evaluate the therapeutic effect and safety of different doses of TwHF.

## Author contributions

**Conceptualization:** Liang Gong, Lun Wang, Yihan Cao, Chen Li.

**Data curation:** Liang Gong, Lun Wang, Yihan Cao, Chen Li.

**Funding acquisition:** Chen Li.

**Methodology:** Liang Gong, Lun Wang, Yihan Cao, Chen Li.

**Project administration:** Liang Gong, Lun Wang, Yihan Cao, Chen Li.

**Supervision:** Chen Li.

**Visualization:** Liang Gong, Lun Wang, Yihan Cao.

**Writing – original draft:** Liang Gong, Lun Wang, Yihan Cao, Chen Li.

**Writing – review & editing:** Liang Gong, Lun Wang, Yihan Cao, Chen Li.
